# Bioelectrical Impedance Analysis as a Contemporary Biomarker of Obesity in Adults with Marfan- or Loeys-Dietz-Syndrome

**DOI:** 10.31083/j.rcm2306215

**Published:** 2022-06-15

**Authors:** Sebastian Freilinger, Mathieu N. Suleiman, Gert Bischoff, Peter Ewert, Annika Freiberger, Michael Huntgeburth, Ann-Sophie Kaemmerer, Judith Schopen, Christian Meierhofer, Nicole Nagdyman, Harald Kaemmerer, Michael Weyand, Frank Harig

**Affiliations:** ^1^Clinic for Congenital Heart Disease and Pediatric Cardiology, German Heart Centre Munich, Technical University Munich, 80636 Munich, Germany; ^2^Department of Sport and Health Sciences, Technical University Munich, Munich, 80992 Munich, Germany; ^3^Department of Cardiac Surgery, University Hospital Erlangen, Friedrich-Alexander-University, 91054 Erlangen, Germany; ^4^Zentrum für Ernährungsmedizin und Prävention (ZEP), Krankenhaus Barmherzige Brüder München, 80639 Munich, Germany; ^5^Deutsches Zentrum für Herz-Kreislauf-Forschung (DZHK), Munich Heart Alliance, 80636 Munich, Germany

**Keywords:** adults with congenital heart disease, Marfan-Syndrome, Loeys-Dietz-Syndrome, body composition, obesity, bioelectrical impedance analysis

## Abstract

**Background:**

It is clinically widely overlooked that many patients with Marfan- (MFS) or Loeys-Dietz-Syndrome (LDS) are obese. While anthropometric routine parameters are not very suitable, the modern Bioelectrical Impedance Analysis (BIA) seems superior for the acquisition of reliable noninvasive assessment of body composition of patients. The aim of the study was to assess the body composition of patients with MFS/LDS by BIA in order to detect occult obesity, which may be a risk marker for aortic or vascular complications.

**Methods:**

In this exploratory cross-sectional study, 50 patients (66% female; mean age: 37.7 ± 11.7 [range: 17–64] years) with a molecular genetic (n = 45; 90%) or clinical (n = 5; 10%) proven diagnosis of MFS or LDS were enrolled between June 2020 and February 2022. All BIA-measurements were performed with the Multifrequence-Impedance-Analyzer Nutriguard-MS (Data Input, Poecking, Germany).

**Results:**

The MFS/LDS collective was significantly different from an age-, sex-, and BMI-adjusted control in terms of body fat, percent cellularity, body cell mass, extra cellular mass/body cell mass index, and phase angle (all *p *< 0.05). The mean BIA-measured bodyfat was 31.7 ± 8.7% [range: 9.5–53.5%], while the mean calculated BMI of the included patients was 23.0 ± 4.8 kg/$m^{2}$ [range: 15.2–41.9 kg/$m^{2}$]. Therefore, using the obesity cut-off values for the body fat percentage of 25% in men and 35% in women, the BIA classifies as many as 28 patients (56.0%) as obese. In contrast only 12 patients (24.0%) were pre-obese, respectively 3 (6.0%) obese by BMI. The significant difference (*p *< 0.001) had an accordance of 42.7%. Overall, 15 patients (13 MFS; 2 LDS) had previous aortic surgery (n = 14) and/or interventional treatment (n = 2) for aortic complications (aneurysm, aortic dissection). 11 out of these 15 (73.3%) were currently classified as obese by BIA.

**Conclusions:**

The fact that many patients with MFS or LDS are obese is widely unknown, although obesity may be associated with impaired vascular endothelial function and an increased risk of cardiovascular complications. Also, in patients with MFS/LDS, BIA allows a reliable assessment of the body composition beyond the normal anthropometric parameters, such as BMI. In the future, BIA-data possibly may be of particular importance for the assessment of the vascular risk of MFS/LDS patients, besides the aortic diameters.

## 1. Introduction 

Marfan syndrome (MFS) is a rare, genetically determined multiorgan disease that affects 0.002% to 0.017% of the population [1, 2, 3]. Affected is the connective tissue throughout the body, including the skeletal, ocular, pulmonary, central nervous and cardiovascular systems.

The diagnosis of MFS is currently established by clinical and/or genetic criteria, determined by international experts, which have been proven to confirm the diagnosis in over 95% of patients (revised Ghent nosology) [4].

In addition, Marfan syndrome also has concealed features that are not so well-known and are not listed in the Ghent nosology [5].

Loeys-Dietz syndrome (LDS) is also a connective tissue disorder, caused by mutations in the *TGFBR1*, *TGFBR2*, *TGFB2*, *TGFB3*, *SMAD3* and *SMAD2* genes and typically associated with cardiovascular changes and skeletal involvement [6]. More than 95% of affected individuals have aortic root dilatation and are at increased risk for aortic dissection. The diagnosis can be established by molecular genetics with evidence of a pathogenic variant in one of the mentioned genes if present in combination with aortic root dilatation or type A dissection, or if there is additional systemic involvement with characteristic craniofacial, vascular, skeletal or cutaneous manifestations [7].

The most severe and sometimes life-threatening complications in MFS and LDS arise from aortic aneurysm, dissection or even rupture. Therefore, all patients with MFS and LDS need lifelong follow-up and regular assessment of their cardiovascular structures [8].

Currently, most efforts have been directed at preventing these complications. The measures chosen for this purpose are focused on medical prophylaxis of aortic dissection using β- or Angiotensin (AT) blockers. In addition, patients receive prophylactic aortic replacement at a stage when the risk of aortic dissection still appears to be low. It should be emphasized, however, that this measure is not infrequently too late, as up to 10% of patients with MFS experience aortic dissection with “normally” wide aortas. The situation in LDS patients is similar to patients with MFS, although the risk of complications is considered to be even higher [9].

To date, there are only few approaches beyond measuring aortic diameters, assigning specific molecular genetic patterns, or considering family history as factors in patients with MFS or LDS with a particular high risk of aortic dissection.

There is growing evidence that obesity, often already present in childhood and adolescence, is associated with serious adverse health outcomes later in life, including an increased risk of cardiovascular disease, metabolic syndrome disability and premature mortality and is also increasing health costs [10, 11, 12, 13, 14, 15, 16, 17]. In addition, obese individuals are more predisposed to developing acute aortic dissection (AAD) compared to the healthy counterparts and an increase in the number of obese patients appearing with acute Stanford type A aortic dissection (ATAAD) has been observed [17].

However, the recognition of obesity is not always trivial and Bioelectrical impedance analysis (BIA) is a convenient method for assessment of body composition [18, 19]. The commonly used body mass index (BMI) cannot differentiate between fat and lean mass, which may have different effects on health status, and is therefore of limited value in assessing body composition [17, 20, 21].

The aim of the present study was to systematically investigate body composition and especially obesity in a large sample of patients with MFS and LDS and to compare the results with those of healthy controls.

This study should encourage clinicians to assess the nutritional status of patients with MFS and LDS and, based on this, potentially initiate health-promoting interventions, and lifestyle changes to identify and eliminate potential risk factors.

## 2. Materials and Methods

### 2.1 Study Cohort

This clinical study was a joint project of the Clinic for Congenital Heart Disease and Pediatric Cardiology, German Heart Center Munich, Technical University Munich, Munich, Germany and the Department for Cardiac Surgery, Friedrich-Alexander-University Erlangen-Nürnberg, Erlangen, Germany. Patient data was compared with an age-, sex-, and BMI-adjusted healthy control population from a German representative norming sample, including over 200,000 participants [22, 23, 24], implemented in the BIA-Software (NutriPlus© 6.0 Data Input Gmbh, Pöcking, Bavaria, Germany) [24].

### 2.2 Subjects and Measurements

This explorative, cross-sectional study included 50 patients with proven Marfan- or Loeys-Dietz-Syndrome who were admitted between June 2020 and February 2022. The diagnosis of MFS and LDS was established according to the current guidelines, considering the results of a comprehensive clinical examination, multiple imaging modalities and family history. For MFS, the revised Ghent nosology of 2010 was applied [4]. Advanced genetic testing was used for the molecular confirmation of MFS or alternative diagnoses.

For LDS, no formal diagnostic criteria have yet been developed. The diagnosis was established in our study according to Schepers *et al*. [25] in individuals with a heterozygous pathogenic variant in *SMAD2*, *SMAD3*, *TGFB2*, *TGFB3*, *TGFBR1*, or *TGFBR2* who exhibit either an aortic root enlargement, a type A dissection, or compatible systemic features including characteristic craniofacial, skeletal, cutaneous, and/or vascular manifestations found in combination. Patients were included consecutively in the order they presented at the institution and were not selected in prior. Inclusion criteria for the present study were (I) a confirmed diagnosis of Marfan- or Loeys-Dietz-Syndrome, and (II) an age >17 years. The clinical and/or molecular genetic diagnosis of Marfan syndrome (Q87.4 according to ICD-10- GM) was obtained by an experienced Marfan-/LDS specialist. Exclusion criteria were (I) the presence of implanted cardiac devices (pacemakers or “automatic implantable cardioverter defibrillator” (AICD) or prostheses, (II) pregnancy, (III) lack of cognitive competence to consent to research, and (IV) refusal to consent. Medical records were reviewed for patient demographics and clinically relevant data. An appropriate form was completed that included clinical diagnoses, anthropometric and clinical parameters (age, sex, weight, height, Body Mass Index, molecular genetic test results, medication, and data of previous aortic surgery). Weight and height were measured, by health professionals prior to the BIA, with minimal clothing and barefoot, using a calibrated weight scale and stadiometer, respectively.

### 2.3 Bioelectrical Impedance Analysis (BIA)

The BIA was performed with the multifrequency impedance analyzer “Nutriguard MS” from Data Input GmbH, Pöcking, Germany. Basal metabolic rate, body water, fat-free mass (FFM = BCM + ECM), extracellular mass (ECM; interstitium, bone, connective tissue), body cell mass (BCM; muscle and organ cell mass), ECM/BCM-index, cell-percentage (proportion of BCM in fat-free mass), body fat (in kg and%), and the phase angle ϕ (quality of fat-free mass) were evaluated. The “Nutriguard MS” is a validated tool for the assessment of body composition [18]. A single BIA was performed in each patient. Analysis lasted 15 s, and the obtained results were recorded electronically.

For measurement, a sinusoidal alternating current of 0.8 mA at a frequency of 50 kHz is passed through the body via four surface electrodes. Subjects were asked to remove all metal objects (e.g., watches, jewelry) prior to lying supine and barefoot on a consultation bed, with limbs positioned slightly away from the body. After cleaning the skin with a hydroalcoholic solution, one electrode is placed on the imaginary line passing through the styloid process of the radius and the head of the ulna, one on the imaginary line following the second and third metacarpophalangeal joints, one on the imaginary line following the second and third metatarsophalangeal joints, and one along the imaginary line between lateral and medial malleolus.

For classification of obesity, the WHO body fat percentage cut-off values of 25% in men and 35% in women were used [26].

### 2.4 Statistical Analysis

The data analysis was performed using SPSS 28.0 (IBM Inc., Armonk, NY, USA). All statistical evaluations of the data were pseudonymized and not person related.

Descriptive statistical methods were used for data analysis and initial characterization of the study population. Differences between the groups were checked and evaluated using T-, respectively Mann-Whitney-U-Tests. Continuous data was expressed as mean ± standard deviation, categorical or interval scaled variables as absolute numbers or percentages. All occurring *p*-values and tests for significance were performed two-sided. A *p*-value < 0.05 was considered significant.

## 3. Results

### Study Sample, Patient Characteristics and Demographic Data

A total of 50 patients were included, 41 subjects were diagnosed molecular-genetically (n = 36) or clinically (n = 5) as Marfan syndrome according to the revised Ghent criteria [4] and 9 patients were molecular-genetically diagnosed as Loeys-Dietz-Syndrome.

The mean age of all patients at the time of the survey was 37.7 ± 11.7 years [range: 17–64 years]. Most patients were in their second (n = 12; 24%), third (n = 12; 24%) and fourth (n = 15; 30%) decade of life. Three patients (6%) were younger than 20 years, eight older than 50 years (16%). In terms of sex distribution, 33 patients (66%) were female (Table 1). Mean height was 182.7 ± 9.4 cm, 178.5 ± 7.2 cm in female patients and 191.0 ± 7.6 cm in male patients, respectively.

**Table 1.** **Patient demographics**.

Parameter	Overall (N = 50)	MFS (n = 41)	LDS (n = 9)
Age (years)	37.7 ± 11.7 (17–64)	37.3 ± 11.8 (18–64)	39.4 ± 11.7 (17–54)
Sex (F:M)	33:17	27:14	6:3
Height (cm)	182.7 ± 9.4 (160–203)	184.0 ± 9.3 (160–203)	176.9 ± 7.9 (165–189)
Weight (kg)	76.9 ± 16.7 (47–145)	77.3 ± 17.6 (47–145)	75.1 ± 12.2 (63–94)
BMI (${\mathrm{kg/m}}^{2}$)	23.0 ± 4.8	22.8 ± 4.9	24.1 ± 4.1
Moleculargenetically confirmed	45	36	9
Angiotensin-Blocker	24	20	4
Beta-Blocker	30	24	6
No medication*	7	4	3
Arterial hypertension	9	8	1
Sleep apnea syndrome	2	2	-
Hyperlipidaemia	4	4	-
Diabetes mellitus Type I	1	1	-
Dissection	5	4	1
Aortic Surgery	15	13	2

F, female; M, male; N/n, absolute number; MFS, Marfan syndrome; LDS, Loeys-Dietz-Syndrom, *against medical recommendation.

Details regarding comorbidities such as hypertension, dyslipidemia, sleep apnea syndrome, diabetes mellitus and medications are given in Table 1. There was no significant difference between MFS- and LDS-syndrome regarding age, weight, and Body Mass Index (Table 1). Regarding height MFS patients were significantly taller than LDS-patients (184.0 ± 9.3 cm vs. 176.9 ± 7.9 cm; *p* = 0.033).

Also compared to an age-, sex- and Body Mass Index-matched healthy control population, the studied MFS/LDS patients were significantly taller (*p *< 0.05). The data from the BIA are given in Table 2a,2b,2c. There was no significant difference between MFS- and LDS-patients regarding BIA-parameters. Women with MFS/LDS have a higher ECM/BCM index compared to men, indicating a higher proportion of ECM. In addition, women have a higher body fat percentage due to their physiology. Consequently, men with MFS/LDS have higher BCM and percent cellularity compared to women.

**Table 2a.** **Comparison of BIA-Parameters of the overall MFS/LDS collective and the healthy control, by sex**.

Parameter	Overall (LDS + MFS)	Matched Controls	*p*-value	Overall (LDS + MFS) Males	Matched Male Controls	*p*-value	Overall (LDS + MFS) Females	Matched Female Controls	*p*-value
	(N = 50)	(N = 50)		(n = 17)	(n = 17)		(n = 33)	(n = 33)	
Phase angle (°)	5.3 ± 0.7	6.9 ± 0.3	< 0.001*	5.9 ± 0.6	6.9 ± 0.3	<0.001*	5.0 ± 0.6	6.9 ± 0.3	< 0.001*
Total Body Water (L)	37.9 ± 7.1	43.0 ± 6.5	0.004*	44.5 ± 6.5	43.5 ± 6.8	0.728	34.5 ± 4.6	41.3 ± 6.3	< 0.001*
FFM (kg)	51.9 ± 9.8	57.6 ± 8.7	0.003*	61.2 ± 8.6	60.1 ± 8.8	0.772	47.1 ± 6.3	56.4 ± 8.6	< 0.001*
ECM (kg)	26.6 ± 4.1	26.5 ± 3.3	**0.864**	29.6 ± 4.2	27.4 ± 3.3	0.145	25.1 ± 3.1	26.0 ± 3.3	0.261
BCM (kg)	25.3 ± 6.3	31.1 ± 5.5	< 0.001*	31.6 ± 5.2	32.6 ± 5.6	0.333	22.0 ± 3.9	30.4 ± 5.4	< 0.001*
ECM/BCM-Index	1.1 ± 0.1	0.8 ± 0.2	0.005*	1.0 ± 0.1	0.8 ± 0.1	0.017*	1.2 ± 0.2	0.9 ± 0.1	< 0.001*
percent cellularity	48.2 ± 4.0	53.8 ± 1.6	< 0.001*	51.5 ± 3.2	54.2 ± 1.7	0.012*	46.6 ± 3.3	53.7 ± 1.5	< 0.001*
Body fat (%)	31.7 ± 8.7	13.8 ± 2.3	< 0.001*	24.5 ± 6.1	13.8 ± 2.5	< 0.001*	35.4 ± 7.4	13.8 ± 2.1	< 0.001*

N/n, absolute number; MFS, Marfan syndrome; LDS, Loeys-Dietz-Syndrome; FFM, Fat-free Body Mass; ECM, extracellular mass (interstitium, bone, connective tissue); BCM, body cell mass (muscle and organ cell mass); *, statistically significant finding (*p *< 0.05).

**Table 2b.** **Comparison of BIA-Parameters of the MFS collective and their matched healthy controls, by sex**.

Parameter	Overall MFS	Matched Controls	*p*-value	MFS Males	Matched Male Controls	*p*-value	MFS Females	Matched Female Controls	*p*-value
	(N = 41)	(N = 41)		(n = 14)	(n = 14)		(n = 27)	(n = 27)	
Phase angle (°)	5.3 ± 0.72	6.9 ± 0.4	< 0.001*	5.8 ± 0.6	6.9 ± 0.4	<0.001*	5.0 ± 0.4	6.9 ± 0.3	< 0.001*
Total Body Water (L)	37.9 ± 7.4	41.5 ± 6.5	0.012*	44.8 ± 6.5	43.7 ± 6.4	0.751	34.4 ± 6.5	40.3 ± 6.3	< 0.001*
FFM (kg)	51.8 ± 10.1	56.9 ± 8.7	0.007*	61.1 ± 8.9	60.7 ± 8.0	0.905	47.0 ± 6.7	55.1 ± 8.7	< 0.001*
ECM (kg)	26.7 ± 4.4	26.2 ± 3.4	**0.298**	29.9 ± 4.5	27.6 ± 3.1	0.160	25.0 ± 3.3	25.6 ± 3.3	0.613
BCM (kg)	25.1 ± 6.3	30.7 ± 5.5	< 0.001*	31.2 ± 5.3	31.2 ± 5.3	0.505	22.0 ± 4.0	29.6 ± 5.4	< 0.001*
ECM/BCM-Index	1.1 ± 0.2	0.8 ± 0.2	0.005*	1.0 ± 0.1	0.8 ± 0.1	0.011*	1.2 ± 0.2	0.9 ± 0.05	< 0.001*
percent cellularity	48.1 ± 3.9	53.8 ± 1.5	< 0.001*	51.0 ± 3.3	54.4 ± 1.7	0.006*	46.6 ± 3.4	53.5 ± 1.4	< 0.001*
Body fat (%)	32.1 ± 8.3	13.9 ± 2.2	< 0.001*	25.5 ± 5.2	13.5 ± 2.3	< 0.001*	35.5 ± 7.5	14.1 ± 2.2	< 0.001*

N/n, absolute number; MFS, Marfan syndrome; FFM, Fat-free Body Mass; ECM, extracellular mass (interstitium, bone, connective tissue); BCM, body cell mass (muscle and organ cell mass); *, statistically significant finding (*p *< 0.05).

**Table 2c.** **Comparison of BIA-Parameters of the LDS collective and their matched healthy controls, by sex**.

Parameter	Overall LDS	Matched Controls	*p*-value	LDS Males	Matched Male Controls	*p*-value	LDS Females	Matched Female Controls	*p*-value
	(N = 9)	(N = 9)		(n = 3)	(n = 3)		(n = 6)	(n = 6)	
Phase angle (°)	5.5 ± 0.88	6.8 ± 0.3	0.003*	6.4 ± 0.5	6.7 ± 0.6	0.751	5.0 ± 0.6	7.0 ± 0.1	< 0.001*
Total Body Water (L)	37.6 ± 6.2	44.5 ± 6.2	0.040*	43.2 ± 7.4	42.2 ± 10.0	0.916	34.8 ± 3.2	45.6 ± 4.2	< 0.001*
FFM (kg)	52.2 ± 8.8	60.6 ± 8.5	0.060	61.5 ± 8.2	57.6 ± 13.7	0.778	47.6 ± 4.4	62.2 ± 5.8	< 0.001*
ECM (kg)	26.3 ± 2.7	27.6 ± 2.9	0.221	28.4 ± 3.3	26.5 ± 5.0	0.736	25.3 ± 1.9	28.2 ± 1.7	0.010*
BCM (kg)	25.9 ± 6.6	33.0 ± 5.7	0.032*	33.1 ± 5.3	31.0 ± 8.7	0.806	22.3 ± 3.2	34.0 ± 4.1	< 0.001*
ECM/BCM-Index	1.0 ± 0.2	0.8 ± 0.2	0.005*	0.9 ± 0.1	0.8 ± 0.1	0.903	1.2 ± 0.2	0.8 ± 0.2	0.002*
percent cellularity	49.0 ± 4.6	54.2 ±1.8	0.007*	53.8 ± 2.3	53.5 ± 2.3	0.895	46.7 ± 3.3	54.5 ± 1.6	< 0.001*
Body fat (%)	30.0 ± 10.6	13.7 ± 2.5	0.002*	19.7 ± 8.9	15.1 ± 3.7	0.458	35.1 ± 7.5	13.0 ± 1.7	< 0.001*

N/n, absolute number; LDS, Loeys-Dietz-Syndrome; FFM, Fat-free Body Mass; ECM, extracellular mass (interstitium, bone, connective tissue); BCM, body cell mass (muscle and organ cell mass); *, statistically significant finding (*p *< 0.05).

Compared to an age-, sex-, and BMI-adjusted healthy control population, the investigated MFS population differed significantly with respect to body fat (*p *< 0.001), body water (*p* = 0.012), fat-free body mass (*p* = 0.007), percent cellularity (*p *< 0.001), BCM (*p *< 0.001), ECM/BCM index (*p *< 0.001), and phase angle (*p *< 0.001). Thereby, percent cellularity, fat-free body mass, total body water, BCM, as well as phase angle were significantly lower, whereas percent body fat, and ECM/BCM-index were significantly higher.

Compared to an age-, sex-, and BMI-adjusted healthy control population, also the LDS patients differed significantly in body fat (*p* = 0.001), BCM (*p* = 0.032), percent cellularity (*p* = 0.007), ECM/BCM index (*p* = 0.05) and phase angle (*p* = 0.003).

Notably, in the total collective of MFS/LDS-patients, the percentage of body fat determined by BIA is 31.7 ± 8.7% [range: 9.5–53.5%]. Using the obesity cut-off values [26] the BIA classified 23 MFS- (56.1%) and 5 of the LDS-patients (55.6%) as obese.

In contrast, based on the Body Mass Index, 11 patients (26.8%) within the Marfan-group and three (33.3%) in the LDS-group were classified as overweight (BMI 25–30), respectively. Further 2 MFS (4.9%) and 1 LDS patient (11.1%) were obese (BMI >30). Interestingly, the Body Mass Index was statistically only in moderate agreement with body fat percentage determined in BIA (Cohen’s Kappa = 0.427; *p *< 0.001) (Table 3).

**Table 3.** **Comparison between BIA and BMI in MDS/LDS study population**.

Parameter	Overall (N = 50)	MFS (n = 41)	LDS (n = 9)
BMI-Classification (in kg/$m^{2}$)	23.0 ± 4.8	22.8 ± 4.9	24.1 ± 4.1
Underweight (<18.5)	7	7	0
Normal (18.5–24.9)	28	22	6
Overweight (25–29.9)	12	10	2
Obese (>30)	3	2	1
BIA-Classification (bodyfat in%)	31.7 ± 8.7	32.1 ± 8.3	30.0 ± 10.6
≤25% (M); ≤35% (F)	22 (9:13)	18 (8:10)	4 (1:3)
>25% (M); >35% (F)	28 (8:20)	23 (6:17)	5 (2:3)

F, female; M, male; N/n, absolute number; MFS, Marfan syndrome; LDS, Loeys-Dietz-Syndrome.

Looking at the aortic history (Table 4), overall, 15 patients (13 MFS; 2 LDS) had previous aortic surgery (n = 14) and/or interventional treatment (n = 2) for aortic complications (aneurysm, aortic dissection). 11 out of these 15 (73.3%) were classified as obese by BIA after surgery. Of five dissections, two patients were obese by BIA (40%). To exclude the possibility that the patients gained weight as a result of the surgery, we determined the preoperative weight from the patients’ operative records. Only three patients had a relevant weight gain after surgery.

**Table 4.** **Overview f different parameter of the operated patients collective**.

Type	Sex	Age at surgery (years)	Acute aortic dissection	Type of surgery/aortic stenting	Age at BIA (years)	Bodyweight [in kg] prior operation	Bodyweight [in kg] at BIA	Body fat (%)
MFS	M	25		Valve Sparing Root Replacement (David Procedure)	25	80	66	16.7
MFS	M	27/39	**X**	1. Conduit 25 mm, Type Sorin Carbon	42	69	74	20.0
				2. Redo surgery: Ao asc. replacement				
MFS	F	28	**X**	Valve Sparing Root Replacement (David Procedure), Aortic stent grafting	35	65	67	29.4
MFS	M	45	**X**	Aortic stent grafting	46	94	94	21.5
MFS	F	29		1. Valve Sparing Root Replacement (David Procedure)	40	68	66	**43.3**
				2. Redo Surgery: Aortic valve replacement, replacement of the remaining ascending aorta and aortic arch				
MFS	F	54	**X**	Replacement Ao asc.-, Reconstruction aortic bulb	55	75	74	**39.7**
MFS	M	35/41		1. Aortic root replacement	42	76	80	**31.1**
				2. Aortic arch replacement				
MFS	M	35		Valve Sparing Root Replacement (David Procedure)	38	95	98	**29.4**
MFS	F	41		Valve Sparing Root Replacement (David Procedure)	48	72	88	**35.6**
LDS	F	41		Valve Sparing Root Replacement (David Procedure)	41	78	78	**41.6**
MFS	F	40		Valve Sparing Root Replacement (David Procedure)	53	109	95	**41.2**
MFS	F	39		Valve Sparing Root Replacement (Yacoub Procedure)	58	82	93	**45.0**
LDS	F	45/49	**X**	1. Aorto-thoracic interponat	54	66	65	**35.1**
				2. Replacement thoracic aorta				
MFS	F	27		Valve Sparing Root Replacement (David Procedure)	36	78	91	**47.2**
MFS	F	44		Valve Sparing Root Replacement (David Procedure)	45	78	78	**38.3**

BIA, Bioelectrical Impedance Analysis; F, female; M, male; N/n, absolute number; MFS, Marfan syndrome; LDS, Loeys-Dietz-Syndrome.

## 4. Discussion

We describe the use of modern Bioelectrical Impedance Analysis (BIA) for the assessment of body composition and to address the relationship between obesity and the risk of aortic complications in patients with Marfan- or Loeys-Diets-syndrome.

Thoracic aortic aneurysm and dissection (TAAD) is a major cause of morbidity and mortality in developed countries [27].

One group of patients at high risk for developing aortic aneurysm and aortic dissection are those with hereditary connective tissue disorders, such as Marfan or Loeys-Dietz syndrome [28]. In this category of patients, aortic dilatation and resulting complications develop from intrinsic molecular genetic alterations of the *FBN1* gene in Marfan syndrome or the *TGFBR1* or *TGFBR2* genes in LDS [27, 29].

Moreover, also isolated peripheral aneurysms have been described in Marfan syndrome in the carotid, subclavian, axillary, internal mammary, ulnar, iliac, and superficial femoral arteries, often aneurysms, which are mostly detected incidentally [5].

The question remains whether histopathological changes in the aorta alone are sufficient to explain occurring aortic complications in MFS or LDS and whether additional pathological mechanisms affecting the vessel walls are responsible.

Aggravating factors are certainly the presence of a bicuspid aortic valve, arterial hypertension and perhaps obesity [30, 31]. About 60–80% of adult patients with MFS develop aortic root dilatation, with a higher prevalence in men than in women [32].

Obesity has long been recognized as an important risk factor that increases cardiovascular morbidity and mortality, as well as health care cost [33, 34, 35]. Moreover, an increasing incidence of acute aortic dissection has been observed in obese patients [17].

Whether the presence of obesity in MFS or LDS patients may also have an impact on the development of aortic aneurysm or aortic dissection is largely undetermined. There is very scattered evidence in the literature that this may be the case [5]. Although patients with Marfan syndrome have historically been considered to have an asthenic body habitus, and are usually tall and slender, Yetman *et al*. [31] classified out of 50 Marfan patients (20 male and 30 female) 11 (n = 22%) as obese, and 18 (36%) patients as overweight or obese, using the BMI according to the criteria of the Centers for Disease Control and Prevention (USA) classification. The median weight and height for the entire patient cohort was 83 kg [range: 50–152 kg] and 180 cm [range: 165–205 cm], respectively, and the mean BMI was 25.4 ± 7.4 kg/$m^{2}$ [31]. He concluded that obesity is common in adults with Marfan syndrome and may be associated with an increased risk of aortic complications [31].

In the present study, all 50 subjects were diagnosed molecular-genetically (n = 45) or clinically (n = 5) as Marfan or LDS syndrome according to the current diagnostic criteria. Their number, mean age and sex distribution were comparable with those of Yetman’s study.

Based on the body mass index, 26.8% of the Marfan patients and 33.3% of the LDS patients were classified as overweight, and 4.9% of MFS and 11.1% of LDS patients were obese, respectively. In his publication, Yetman emphasizes the problem of characterizing obesity in patients with connective tissue disease. This applies in particular to the conventional anthropometric measurements, such as using caliper measurement or measured waist circumference which can result in a false estimation of the body fat because of the percentage cutaneous laxity and altered connective tissue properties of these patients [31].

However, for the assessment of body composition more sophisticated methods are available. Bioelectrical impedance analysis (BIA) is a contemporary, non-invasive exploratory method for determining the body composition of a subject. Since several year, the use of BIA in cardiology and cardiac surgery has considerably increased.

In the field of cardiology, BIA is used for the assessment and treatment of heart failure [36, 37, 38, 39]. In cardiac surgery, BIA can provide predictive evidence of peri-operative/postoperative risk, as well as for treatment management [40]. In the field of congenital cardiology, recent data are available that BIA provides determinants for assessing exercise capacity and cardiac compensatory status, which can be used as prognostic predictors or for therapy management [38, 41].

As an indirect method, BIA uses mathematical equations to calculate the body composition based on the collected measurement parameters. The basis for bioimpedance analysis is the different electrical conductivity of tissues. While electrolyte-containing body water conducts electricity very well, adipose tissue behaves like an insulator. These properties make it possible to use BIA to differentiate tissues and determine a person’s body composition.

Impedance is the frequency-dependent resistance of a conductor to the flow of an alternating electric current. The measure of impedance (Z) is a composite of the two vectors resistance (Rx) and reactance (Xc). Resistance is the pure resistance of a conductor to an alternating current and is inversely proportional to the total body water. Reactance is another variable for calculating body cell mass. It results from the resistance to the flow of an alternating current caused by the capacitive effect of cell membranes, tissue interfaces, and nonionic tissues. Resistance and reactance can be distinguished from each other based on the phase-sensitive electronics of the BIA device. The capacitors of the AC circuit cause a time change Δ t, measured in degrees, and referred to as the phase angle ϕ. The phase angle is directly proportional to the mass of the body cells.

Thus, BIA allows an exact estimation of the fat and muscle mass of the body and the intracellular and extracellular water content inside and outside the cells [40, 42].

The MFS population differed significantly from an age-, sex-, and BMI-matched healthy control population in terms of body fat, body water, fat-free body mass, percent cellularity, BCM, ECM/BCM index, and phase angle. Thereby, percent cellularity, fat-free body mass, total body water, BCM, and phase angle were significantly lower in Marfan patients, while percent body fat, and ECM/BCM index were significantly higher.

LDS patients also differed significantly from an age-, sex-, and BMI-matched healthy control population in terms of body fat, BCM, percent cellularity, ECM/BCM index, and phase angle.

Due to several limitations of BMI in general [43] and especially in the studied patient-group [5], we hypothesize that BIA-derived body fat is a more suitable tool to assess obesity in patients with connective tissue disorders as MFS or LDS. The comparison of both measurements regarding overweight/obesity revealed only a moderate agreement of BMI and BIA (Cohen’s Kappa = 0.427; *p *< 0.001). The actual prevalence of obesity among MFS or LDS could therefore be even higher than previously assumed by Yetman and colleagues. As this study is the first of this kind, these findings have to be investigated and proved in future studies.

Looking at the natural and the postoperative or postinterventional course of the included patients, a total of 15 patients (13 MFS; 2 LDS) had severe aortic complications. Previous aortic surgery for aortic complications (aneurysm, aortic dissection) had been performed in 14, and/or aortic stent grafting in two. Of these 15 patients, 11 (73.3%) were classified postoperatively as obese by BIA.

A similar observation is described by Yetman *et al*. [31] who correlates this observation with the fact that adipose tissue is known to be metabolically active. Adipose tissue can adversely affect aortic histology and biomechanics by the production of several different cytokines and vasoactive substances including angiotensin II and TGF-beta [31]. In his study, other cardiovascular risk factors with potentially negative effects on vascular function, including hyperlipidemia and smoking habits, were related to adverse outcomes on univariate analysis. However, on multivariate analysis, increased BMI outweighed the impact of all other risk factors [31].

Although a causal relationship between obesity and the occurrence of aortic complications cannot be verified, we consider it advisable to counsel affected patients with MFS and LDS regarding the prevention of obesity and to follow these affected individuals particularly carefully in the long-term course.

## 5. Limitations

The present study recruited a remarkably large sample patient with MFD or LDS. However, some limitations must be considered when interpreting the current results.

Left unconsidered is a widely unknown condition called occult and sarcopenic obesity, which is characterized by average or near-average body weight combined with high body fat. Also a distinction between visceral fat tissue and subcutaneous fat tissue is not reliably possible with the BIA device. It has to be considered that, in most cases, BIA was performed after surgery for aortic complications. Therefore, a direct link between obesity and likelihood for the need of surgery remains debatable. Moreover, it was not the intention of the present study to detect a relationship between obesity and specific current aortic diameters. This would be the subject of a subsequent, independent analysis. Even though modern BIA is a valid and representative method for determining body composition in humans, there are no validation studies on patients with hereditary connective tissue disorders. The transferability to such a patient population can therefore not yet be clearly confirmed.

The sample of patients seen at tertiary care centers do not represent the typical population of patients with MFS or LDS seen by a general practitioner, internist or by a general cardiologist. The prevalence of more severe forms of MFS or LDS in these institutions is likely to be higher than either in community-based hospitals or even in departments for cardiology.

Lastly, the presented data derive solely from patients living in Germany. Generalization of the conclusions and transmission to patients living in other countries or different culture groups is debatable.

## 6. Conclusions

The fact that many patients with MFS or LDS are obese is widely unknown, although obesity may be associated with impaired vascular endothelial function and an increased risk of cardiovascular complications. Also, in patients with MFS/LDS, modern BIA allows a reliable assessment of the body composition beyond the normal anthropometric parameters, such as BMI. In the future, BIA-data may be of particular importance for the assessment of the vascular risk of MFS/LDS patients, besides the aortic diameters.

An experienced routine follow-up care by specialized physicians or centers is imperative for all patients with MFS and LDS. Within this framework, we consider it advisable to counsel affected patients with MFS and LDS regarding the prevention of obesity and to follow these affected individuals particularly carefully in the long-term course.
